# Genetic and epigenetic changes during the invasion of a cosmopolitan species (*Phragmites australis*)

**DOI:** 10.1002/ece3.4144

**Published:** 2018-06-11

**Authors:** Lele Liu, Cuiping Pei, Shuna Liu, Xiao Guo, Ning Du, Weihua Guo

**Affiliations:** ^1^ Institute of Ecology and Biodiversity College of Life Sciences Shandong University Jinan China; ^2^ College of Landscape Architecture and Forestry Qingdao Agricultural University Qingdao China

**Keywords:** DAN methylation, epigenetics, methylation‐sensitive amplification polymorphism, *Phragmites australis*, plant invasion

## Abstract

While many introduced invasive species can increase genetic diversity through multiple introductions and/or hybridization to colonize successfully in new environments, others with low genetic diversity have to persist by alternative mechanisms such as epigenetic variation. Given that *Phragmites australis* is a cosmopolitan reed growing in a wide range of habitats and its invasion history, especially in North America, has been relatively well studied, it provides an ideal system for studying the role and relationship of genetic and epigenetic variation in biological invasions. We used amplified fragment length polymorphism (AFLP) and methylation‐sensitive (MS) AFLP methods to evaluate genetic and epigenetic diversity and structure in groups of the common reed across its range in the world. Evidence from analysis of molecular variance (AMOVA) based on AFLP and MS‐AFLP data supported the previous conclusion that the invasive introduced populations of *P. australis* in North America were from European and Mediterranean regions. In the Gulf Coast region, the introduced group harbored a high level of genetic variation relative to originating group from its native location, and it showed epigenetic diversity equal to that of the native group, if not higher, while the introduced group held lower genetic diversity than the native. In the Great Lakes region, the native group displayed very low genetic and epigenetic variation, and the introduced one showed slightly lower genetic and epigenetic diversity than the original one. Unexpectedly, AMOVA and principal component analysis did not demonstrate any epigenetic convergence between native and introduced groups before genetic convergence. Our results suggested that intertwined changes in genetic and epigenetic variation were involved in the invasion success in North America. Although our study did not provide strong evidence proving the importance of epigenetic variation prior to genetic, it implied the similar role of stable epigenetic diversity to genetic diversity in the adaptation of *P. australis* to local environment.

## 1 INTRODUCTION

To survive and spread successfully in a new environment distinct from their original native range, invasive plants need phenotypic variation and plasticity in order to adapt to the new habitat (Davidson, Jennions, & Nicotra, 2011), although a few invaders appear preadapted (Dlugosch & Parker, 2007; Schlaepfer, Glättli, Fischer, & van Kleunen, 2010). Some species could obtain more genetic diversity through multiple introductions and hybridization during the invasion process (Genton, Shykoff, & Giraud, 2005; Kelager, Pedersen, & Bruun, 2013; Rosenthal, Ramakrishnan, & Cruzan, 2008; Suehs, Affre, & Médail, 2004), but other invasive species, especially clonal plants, may maintain low levels of genetic diversity after gene drift accompanied by invasion (Hollingsworth & Bailey, 2000; Lambertini et al., 2010; Lindholm et al., 2005; Loomis & Fishman, 2009). While adaptation is accomplished by genetic changes through mutation, drift and selection are very slow for some introduced species, so epigenetic mechanisms can provide an alternative source of ecologically phenotypic diversity for rapid adjustment (Medrano, Herrera, & Bazaga, 2014). Epigenetic diversity can generate massive heritable variation of ecologically relevant plant traits such as root allocation, drought tolerance and nutrient plasticity (Zhang, Fischer, Colot, & Bossdorf, 2013), and it appears to increase the productivity and stability of plant populations in *Arabidopsis thaliana* under artificial conditions (Latzel et al., 2013). An increasing number of studies have also demonstrated the common existence and significant role of epigenetic variation in plant populations of herbs (Foust et al., 2016; Herrera, Medrano, & Bazaga, 2014; Medrano et al., 2014; Preite et al., 2015; Schulz, Eckstein, & Durka, 2014), shrubs (Avramidou, Ganopoulos, Doulis, Tsaftaris, & Aravanopoulos, 2015; Herrera & Bazaga, 2013, 2016), and trees (Guarino, Cicatelli, Brundu, Heinze, & Castiglione, 2015; Gugger, Fitz‐Gibbon, PellEgrini, & Sork, 2016; Lira‐Medeiros et al., 2010; Platt, Gugger, Pellegrini, & Sork, 2015; Sáez‐Laguna et al., 2014) under natural conditions. Therefore, epigenetic variation can be a very important mechanism for invasive plant success in a broad range of environments (Douhovnikoff & Dodd, 2014; Richards, Schrey, & Pigliucci, 2012).

Among all identified epigenetic mechanisms, including histone modifications, DNA methylation, and small noncoding RNAs, DNA methylation is relatively stable with transgenerational heritability that can be independent of heritable genes (Bird, 2007; Eichten, Schmitz, & Springer, 2014), with the result that DNA methylation has attracted the most attention in epigenetic studies in ecology and evolution (Alvarez, Schrey, & Richards, 2015). The pattern of DNA methylation can affect ecologically important phenotypes and plasticity (Herrera & Bazaga, 2013; Nicotra et al., 2015; Zhang et al., 2013) and may play a significant role in adaptation to various habitat conditions (Foust et al., 2016; Richards et al., 2012; Schulz et al., 2014). For example, a naturally occurring epiallele named “NMR19‐4” has been discovered in *Arabidopsis accessions*, and its DNA methylation status is inheritable and independent of genetic variation (He et al., 2018). This epiallele controls leaf senescence and associates with local climates. Moreover, an easy and efficient technique named methylation‐sensitive amplification polymorphism (MS‐AFLP) has been widely used to assess DNA methylation status at a great number of random anonymous loci across the entire genome in nonmodel species without sequenced reference genomes (Alonso, Pérez, Bazaga, Medrano, & Herrera, 2016; Schrey et al., 2013).

*Phragmites australis* (common reed) has a worldwide distribution and has been considered as a model organism for studying plant invasions (Meyerson, Cronin, & Pyšek, 2016) according to the criteria for identifying model organisms in invasion science adapted by Kueffer, Pyšek, and Richardson (2013). Two main introduced lineages in North America have been detected (Guo, Lambertini, Li, Meyerson, & Brix, 2013; Meyerson, Lambert, & Saltonstall, 2010). One, known as Haplotype M (hereafter “INT”), has spread dramatically across much of the North America, especially in the Great Lakes regions (Saltonstall, 2002). The other, represented by Haplotype M1 and I (hereafter “DELTA” and “LAND”), was native to Mediterranean region, sub‐Saharan Africa, and the Middle East and has expanded along the Gulf Coast of the United States and in the northwest of South America (Lambertini, Sorrell, Riis, Olesen, & Brix, 2012; Lambertini, Mendelssohn et al., 2012). The introduced population of *P. australis* had a higher level of genetic diversity and heritable phenotypic variation in its invasive range than in parts of its native range, as multiple and uncontrolled immigration events may have occurred from different European regions to North American (Lavergne & Molofsky, 2007). Some heritable traits and ecophysiological differences in the common reed may contribute to invasion success but hitherto cannot be explained by particular genetic lineages (Mozdzer, Brisson, & Hazelton, 2013). However, very few investigations of natural epigenetic variation of *P. australis* have been reported, and previous studies were conducted just at a small scale such as in midcoast Maine (Spens & Douhovnikoff, 2016) and in the Songnen Prairie of China (Qiu, Jiang, & Yang, 2016). The understanding of potential epigenetic mechanisms in the invasion of *P. australis* is still very limited.

In this study, we first compared patterns of genetic and epigenetic variation in *P. australis* grown in a common garden collected from around the globe using AFLP and MS‐AFLP methods. We collected samples from native and introduced groups in North America and samples of *P. australis* from the native geographic regions from where the introduced groups were believed to have originated, in order to be able to detect the direction and the degree of genetic and epigenetic changes during the invasion of *P. australis*. We tested the following hypotheses: (1) introduced groups increased epigenetic diversity to compensate for the loss of genetic diversity in response to heterogeneous environments, and (2) introduced groups could acquire epigenetic variation similar to that of the native groups faster than genetic variation. In a word, we would expect rapid epigenetic adaptation of introduced groups to new environments before slow genetic accommodation occurs.

## 2 MATERIALS AND METHODS

### 2.1 Plant material

All samples were collected in a common garden at Fanggan Research Station of Shandong University in Shandong Province, China (36°26′N, 117°27′E), where they had been growing for 2 years. This station has a typical warm temperate monsoon climate with a hot rainy summer and a cold dry winter under climate regulation effects of forest (Sun, Li, Guan, Liu, & Zhang, 2017). To avoid developmental epigenetic variation, all leaf samples for molecular analyses were picked at the same position of the plant before flowering phase. A total of 75 specimens of *P. australis*, representing eight phylogeographic groups, were sampled to investigate genetic and epigenetic variation (Table 1). The invasive *P. australis* (INT) in the Great Lakes region was likely introduced from the native European counterpart (EU) based on haplotype evidence, and hybridization between the native and invasive groups in nature has not yet been documented in North America (Saltonstall, 2002, 2003; Saltonstall, Lambert, & Rice, 2016). The LAND and DELTA groups of *P. australis* are two of five identified phenotypes growing sympatrically in the Gulf Coast region. The DELTA group is a typical introduction from the Mediterranean region (MED) and has become one predominant lineage in the Gulf Coast region (Lambertini, Sorrell et al., 2012). The origin of the LAND type is still debated, and we treat this type as a native group in our study due to the following reasons: (1) LAND type has existed for a longer time than DELTA type as the genetic evidence supports an ancient introduction for LAND type and a recent introduction for DELTA type (Lambertini, Sorrell et al., 2012), (2) LAND type is not invasive in this area with only scattered occurrences, and (3) we just used LAND type as a reference for the introduced DELTA group, which coexisted under the homogeneous environment of the Gulf Coast region. *Phragmites mauritianus* may be a hybrid of LAND type origin (Lambertini, Sorrell et al., 2012). Individuals from Australia (FEAU) were also analyzed as *P. mauritianus* from Tropical Africa (TA) as an outgroup.

**Table 1:** Samples from different regions around the word

Group	Origin	Country	Sample size	Ploidy level
NAT	Great Lakes	United States and Canada	6	4
INT	Great Lakes	United States and Canada	13	4
LAND	Gulf Coast	United States	13	4,6
DELTA	Gulf Coast	United States	6	4,6
EU	Europe	Denmark, Italy, etc.	11	4,8
MED	Mediterranean	Italy, Algeria, etc.	11	4
FEAU	Far East/Australia	Australia	11	8,10
TA	Tropical Africa	Uganda	4	—

### 2.2 AFLP and MS‐AFLP analysis

Genomic DNA was extracted from fresh leaf tissue according to the cetyltrimethylammonium bromide (CTAB) method (Doyle & Doyle, 1987). The yield and quality of extracted DNA were determined with both 0.8% agarose gels and a microscope spectrophotometer. We investigated all specimens for genetic and epigenetic variation with AFLP and MS‐AFLP methods. The method of MS‐AFLP was adapted from a standard AFLP, replacing the *Mse*I enzyme in two separate runs with the methylation‐sensitive enzymes *Hpa*II and *Msp*I using appropriate adaptors and primers. The AFLP and MS‐AFLP protocols were used referring to Schulz et al.(2014). Fragment analysis was performed on an ABI3730XL DNA capillary sequencer (Applied Biosystems, Foster City, USA) with a Rox‐500 internal size standard (Applied Biosystems) in Shandong Academy of Agricultural Sciences, and then the AFLP and MS‐AFLP fragment profiles were scored with PEAK SCANNER v1.0 (Applied Biosystems). Both AFLP and MS‐AFLP marker systems utilized three selective primer combinations. The digestion and PCR were repeated in part of the samples (16, approximately 21% of all) with all six pairs of primers to verify the reliability of used bands.

### 2.3 Statistical analysis

To determine the DNA methylation status of every locus from the fragment presence/absence scores of both *Eco*RI‐*Msp*I and *Eco*RI‐*Hpa*II reactions, the R‐based statistical package “msap” was carried out (Pérez‐Figueroa, 2013). In the selected scoring strategy, the presence of both *Eco*RI‐*Msp*I and *Eco*RI‐*Hpa*II bands is an unmethylated state, and the presence of only one band, either *Eco*RI–*Hpa*II or *Eco*RI–*Msp*I, represents a methylated state. However, the absence of both *Eco*RI–*Hpa*II and *Eco*RI–*Msp*I bands may reveal either the full methylated state or genetic variation. As these samples were from different ramets with expected high genetic diversity and we cannot ignore the possibility of genetic change in the specific locus, so we adopted the approach considering the absence of both *Eco*RI‐*Hpa*II and *Eco*RI‐*Msp*I bands as an uninformative state (i.e., missing data) with the function msap (no.bands = “u”). Then, we screened methylation‐susceptible loci (MSL), for which the observed proportion of methylated status across all samples exceeded the error rate‐based threshold (5% by default).

The “msap” package also provides a report on methylation levels for each group, but it is not forthright to estimate the global level of individual methylation. It is unambiguous that the presence in both bands denotes an unmethylated state, the presence in only *Eco*RI–*Hpa*II denotes a hemimethylated state in external cytosine, and the presence in only *Eco*RI‐*Msp*I denotes a full‐ or hemimethylated state in internal cytosine, but no consensus exists on the interpretation of the last pattern where neither band is present. The last pattern caused by full methylation or genetic mutation in the target site should be considered as full methylated data, null data, or missing data. Here, all three strategies were used to compare global cytosine methylation percentage among ploidy levels in common reed. The global methylated cytosine was established using the sum of the internal methylated and the external hemimethylated cytosines.

The genetic and epigenetic diversity of every population was determined using the population genetic software GenAlEx 6.5 (Peakall & Smouse, 2012) in Microsoft Excel to calculate Shannon's Information Index (I), percentage of polymorphic loci (P), and unbiased expected heterozygosity (uHe). Hierarchical AMOVAs were calculated to assess the structure of genetic and epigenetic variation. Pairwise population Φ_ST_ values were used to evaluate genetic and epigenetic differentiation among all groups. In addition, principal component analysis (PCA) was conducted with the package “msap” to compare the variance between native and introduced populations in the Great Lakes region and between two introduced populations in the Gulf Coast region.

## 3 RESULTS

### 3.1 DNA methylation level

**Figure 1:**
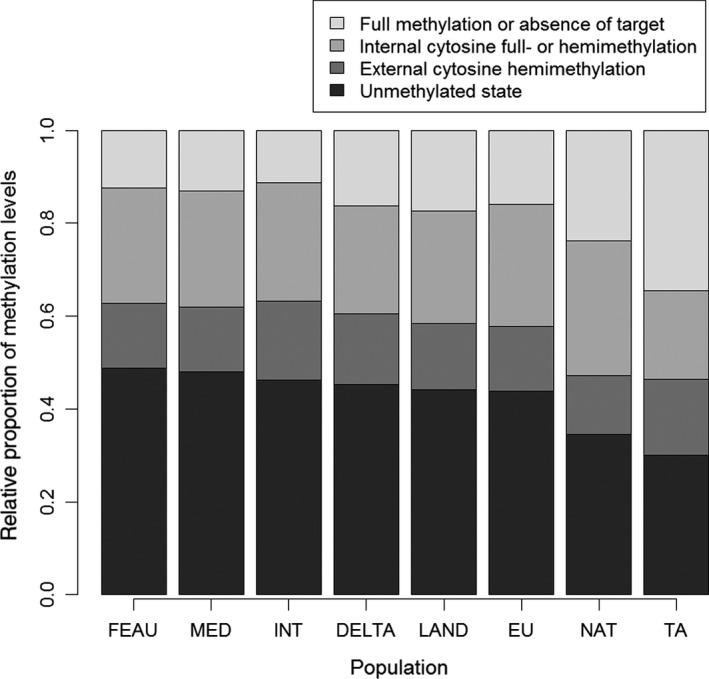
Relative DNA methylation levels in all groups of Phragmites. Among all groups, the percentage of unmethylated states was highest, and internal cytosine methylations were more frequent than external cytosine hemimethylation. The NAT group (native population in the Great Lakes region of *Phragmites australis*) and the PM group (*Phragmites mauritianus*) held substantial states of full methylation or absence of target

**Figure 2:**
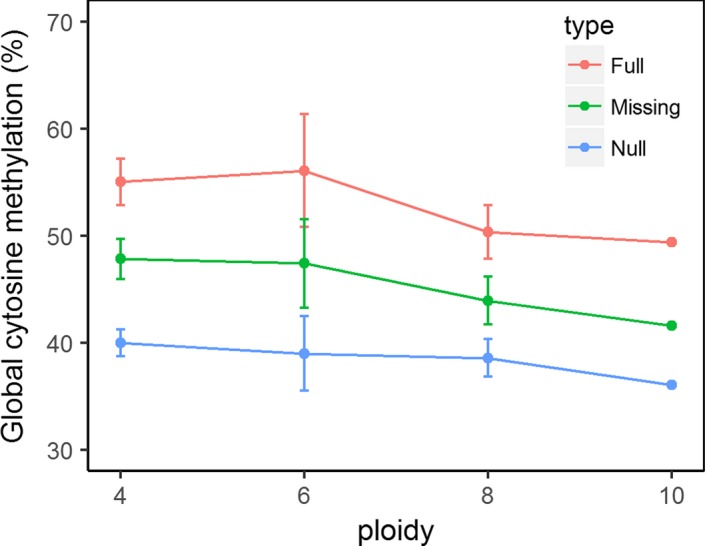
Global cytosine methylation in *Phragmites australis* leaves from different ploidy levels. Each dot denotes the group mean, bars indicate ± standard error (*SE*), and colors indicate different scoring methods for treating the last band pattern of MSAP. The global cytosine methylation did not differ significantly across ploidy levels

Similar DNA methylation levels among all groups were determined through the MS‐AFLP markers except for the NAT and TA groups, which exhibited a relatively high level of uninformative state (i.e., full methylation or absence of target) (Figure 1). The DNA methylation levels of each group calculated from the MSL data revealed that the unmethylated sites were the most common among all populations of *P. australis*, followed by internal cytosine methylated and hemimethylated, and uninformative sites. In addition, we did not detect significant differences across ploidy levels in our samples of *P. australis* when we observed these uninformative loci as full methylated (ANOVA: *p* = .607), missing (ANOVA: *p* = .645), or null data (ANOVA: *p* = .897) (Figure 2).

### 3.2 Genetic and epigenetic diversity

We obtained 154 polymorphic AFLP loci and 151 MSL loci, and diversity indices showed high levels of both genetic and epigenetic variation (Table 2). Among all groups, LAND had the highest level of genetic diversity while EU had the highest level of epigenetic diversity. Almost all groups of *P. australis* displayed a higher value of all three diversity indices for epigenetic than genetic variation except for LAND where uHe for genetic variation (uHe = 0.40) was slightly higher than that for epigenetic variation (uHe = 0.39).

**Table 2:** Genetic and epigenetic diversity of *Phragmites australis*

Pop	I‐gen	%P‐gen	uHe‐gen	I‐epi	%P‐epi	uHe‐epi
DELTA	0.388	0.623	0.296	0.460	0.841	0.344
EU	0.475	0.786	0.344	0.523	0.954	0.375
FEAU	0.397	0.656	0.288	0.443	0.861	0.312
INT	0.359	0.623	0.255	0.469	0.874	0.330
LAND	0.477	0.825	0.340	0.480	0.921	0.339
MED	0.385	0.630	0.280	0.487	0.940	0.343
NAT	0.241	0.429	0.178	0.312	0.530	0.240
TA	0.421	0.766	0.322	0.352	0.583	0.294
Total	0.393	0.670	0.288	0.441	0.813	0.322

In the Great Lakes region, the introduced group (INT) exhibited levels of both genetic and epigenetic diversity higher than the native group (NAT) but lower than original groups (EU). Moreover, the native group had the lowest genetic and epigenetic diversity among all groups, and the introduced group followed.

In the Gulf Coast region, the native group (LAND) had a much higher level of genetic diversity than the introduced group (DELTA) but a lower level of epigenetic diversity using the index uHe. The DELTA group had comparable genetic and epigenetic diversity with one possible original group (MED). Compared with another original group, the DELTA group had lower genetic diversity but higher epigenetic diversity.

### 3.3 Genetic and epigenetic structure

AFLP and MS‐AFLP revealed similar variances among and within groups (see Table 3). Most of the genetic and epigenetic variation existed within rather than among groups, but genetic variance among groups (Φ_ST_ = 0.182, *p* = .001) was more than epigenetic variance (Φ_ST_ = 0.072, *p* = .001).

**Table 3:** Pairwise population Φ_ST_ values of genetic (above) and epigenetic (below) variation

DELTA	EU	FEAU	INT	LAND	MED	NAT	TA	
—	0.009	0.092*	0.053*	0.081*	0.000	0.259*	0.286*	DELTA
0.062*	—	0.166**	0.021	0.123**	0.039**	0.268**	0.295*	EU
0.034*	0.067*	—	0.228**	0.183**	0.129**	0.337**	0.346*	FEAU
0.008	0.029*	0.084**	—	0.198*	0.111**	0.356**	0.388**	INT
0.000	0.060*	0.088**	0.105**	—	0.130**	0.204**	0.230*	LAND
0.103*	0.018	0.046**	0.052**	0.039*	—	0.291**	0.374*	MED
0.062	0.138**	0.196**	0.191**	0.145*	0.144**	—	0.334*	NAT
0.062*	0.079*	0.113*	0.167**	0.116*	0.114*	0.163*	—	TA

9,999 permutations, *p < .05, **p < .001.

**Figure 3:**
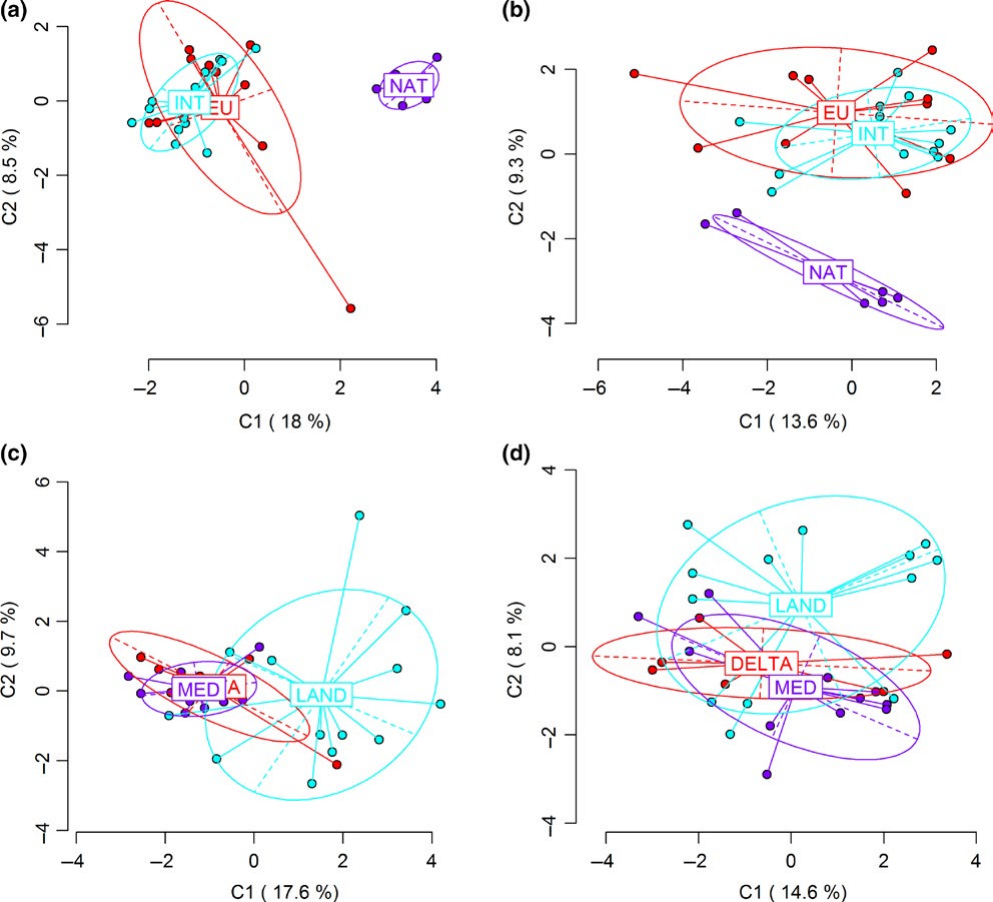
Principal component analysis showing the genetic (a,c) and epigenetic (b,d) variation among several groups in the Great Lakes region (a,b) and the Gulf Coast region (c,d). NAT = the native group in the Great Lakes region; INT = the introduced group in the Great Lakes region; Eu = the noninvasive group in Europe; LAN = the land‐type group in the Gulf Coast region; DELTA = the delta‐type group in the Gulf Coast region; MED = the noninvasive group in the Mediterranean area. Both genetic and epigenetic evidence indicated INT group was similar to EU group (a,b) and DELTA group was similar to MED group (c,d).However, INT group was not closer significantly to NAT group in epigenetic variation (b) relative to genetic variation (a), and DELTA group did not significantly tend toward LAND group in epigenetic variation (c) relative to genetic variation (d)

In the Great Lakes region, the introduced group INT showed a very low level of genetic (Φ_ST_ = 0.021) and epigenetic (Φ_ST_ = 0.029) differentiation from the original group EU. The native group NAT had more genetic variance with the introduced group INT (Φ_ST_ = 0.356) than the original group EU (Φ_ST_ = 0.268), and the NAT group still had more epigenetic variance with the INT group (Φ_ST_ = 0.191) than with EU (Φ_ST_ = 0.138). The PCA showed less difference in epigenetic variation between the INT and NAT group than in genetic variation, but this tendency was not significant [Figure 3a,b].

In the Gulf Coast region, there was low genetic and epigenetic variance (both nearly zero) between the introduced group DELTA and its possible original group MED, and there was a lower level of genetic (Φ_ST_ = 0.081) and epigenetic (Φ_ST_ = 0.008) differentiation between the LAND group and the DELTA group than that between the LAND group and the MED group (Φ_ST_ = 0.130 for genetic variation; Φ_ST_ = 0.039 for epigenetic variation). The TA group had the lowest level of genetic differentiation with the LAND group among all groups of *P. australis*, but not the lowest level of epigenetic differentiation with the LAND group. The PCA displayed a weak decreasing trend of epigenetic differences between the LAND and DELTA group compared with genetic differences [Figure 3c,d].

## 4 DISCUSSION

This is not an in situ study as all samples were collected from a common garden, which must affect the status of some unstable epigenetic variation. Nevertheless, our results are practical and valuable for the following reasons: (1) it is such a short time in the common garden compared to a hundred years of introduced history that most relatively stable epigenetic variation could remain; (2) the common environment has few chances to cause new epigenetic differences among all individuals. In other words, common garden can separate plastic and heritable components of epigenetic variation (Kilvitis et al., 2014; Richards et al., 2012), and samples in our study still maintain most transgenerational epigenetic variation in which ecologists and evolutionary biologists are interested (Verhoeven, vonHoldt, & Sork, 2016). Moreover, the common garden allowed us to control well the developmental stage, which is the other important factor influencing epigenetic variation besides the environmental one.

### 4.1 Massive epigenetic variation is correlated with genetic variation

Consistent with the previous study (Spens & Douhovnikoff, 2016), we found a significant difference in epigenetic variation between introduced and native groups. Furthermore, our result revealed that epigenetic structure showed a positive correlation with genetic structure, and epigenetic (as well as genetic) distance also provided coincident evidence for the identified migration of *P. australis* (Lambertini, Sorrell et al., 2012; Saltonstall, 2002), but epigenetic distance compared with genetic distance was clearly reduced and possibly more related to individual microhabitats. For example, coefficients of genetic differentiation supported the previous haplotype‐based conclusion that *P. mauritianus* contributed to the hybridization of the LAND type of *P. australis* (Lambertini, Sorrell et al., 2012), but the epigenetic evidence in our study cannot (Table 3).

The cytosine methylation level in *P. australis* is relatively steady among geographic and genomic groups, and the detected differences were more due to genetic variation. The reason for the relatively abundant loci with uninformative state in the NAT and TA groups was more likely to be absent from the genetic target rather than full methylation, as the two groups were very different from the other groups in genetic variation (Table 2). Polyploidization is an important evolutionary event in plants and may result in substantial changes in genomewide methylation, but we did not detect the expected increased cytosine methylation with ploidy series (Figure 2). There are very few studies comparing intraspecific DNA methylation levels across wild plants differing in ploidy, and this type of study cannot set apart how much the DNA methylation results were from ploidy versus environmental factors (Alonso, Balao, Bazaga, & Pérez, 2016). MS‐AFLP actually cannot provide a quantitative global DNA methylation forthright (Alonso, Pérez et al., 2016), and the global DNA methylation level in this species should be established by high‐performance liquid chromatography (HPLC) to uncover the role of DNA methylation in natural polyploidizations. Some cytotypes of *P. australis* can naturally coexist but others cannot (Table 1), so ploidy effects are counteracted by the local environment sometimes. However, the ploidy differences within the samples had little effect on genetic and epigenetic changes during natural invasions in this study.

### 4.2 The epigenetic diversity may compensate for limited genetic diversity

Many indices have been developed to describe diversity, and all indices perform congruously in common situations; but they may conflict in some cases. In our study, we calculated three indices to compare the genetic and epigenetic diversity among all groups. When divergence occurred, the uHe index was preferred because of the limited number of individuals from each group (Nei, 1978). Consistent with most previous epigenetic studies of natural population differentiation (Choi, Roy, Park, & Kim, 2016; Foust et al., 2016; Kim, Im, & Nkongolo, 2016; Qiu et al., 2016; Schulz et al., 2014), more epigenetic variance existed within groups than genetic variance. One general explanation for this is that a population with limited genetic diversity, especially after genetic drift, can extend its ecological niche through epigenetic variation, which is potentially sensitive to environmental stimulation (Richards, 2006). As some epigenetic variation is affected by developmental factors instead of the environment, common epigenetic variations within populations could be detected. Therefore, numbers of specific loci with ecological effect can and should be determined in rigorous research through generalized linear models or other statistical methods.

Invasive species can increase their genetic diversity through interbreeding with local or other introduced populations in response to diverse habitat environments (Genton et al., 2005; Lavergne & Molofsky, 2007; Rosenthal et al., 2008). Cross‐pollination experiments have demonstrated that that native and introduced populations of Phragmites can hybridize (Meyerson, Viola, & Brown, 2010), but few natural hybridization cases have been detected between the native and introduced lineages (Saltonstall, Castillo, & Blossey, 2014). In the Gulf Coast region, there may be very strong gene flow within and/or among lineages (Meyerson, Lambertini, McCormick, & Whigham, 2012), which caused a relatively high level of genetic diversity. The LAND and DELTA groups were established by independent colonization events (Lambertini, Sorrell et al., 2012), and the LAND group possessed a higher level of genetic diversity than the DELTA group which suggested introduced DELTA group lost some genetic diversity during the invasion process but obtained little genetic variation through interbreeding with other local lineages. This assumption is also supported by the little genetic and epigenetic differentiation between the DELTA and MED groups. Genetic diversity seems to be a key factor explaining its broad ecological adaptation (Kettenring, McCormick, Baron, & Whigham, 2011; Lambertini, Mendelssohn et al., 2012). Compared with LAND group, the DELTA group can adapt to wetter and slightly more saline habitats. Therefore, there is a conflict between lower genetic diversity and the broader ecological amplitude in DELTA group. As the results showed that the epigenetic diversity in the DELTA was as high as in the LAND group, if not higher, the epigenetic diversity partially explained this contradictory. The finding also indicated that epigenetic variation could compensate for decreased genetic variation after an initialized introduction.

### 4.3 Stable epigenetic differentiation has not occurred

Epigenetic variation in invasive plant populations can contribute to phenotypic variation and plasticity for adaptation in new environments as a fast mechanism during the introduction (Richards et al., 2012; Schulz et al., 2014). In the Great Lakes region, our result predicted a constant threat to the native *P. Australis* in which the native group exhibited a very low level of epigenetic variation, as well as genetic variation, possibly because of very rare hybridization between the native and introduced groups (Saltonstall, 2011). While the introduced group in the Great Lakes regions also had low genetic diversity relative to those in the Gulf Coast regions, this finding suggested that fewer introductions limited interbreeding there. While the introduced group could not obtain increased genetic variation, it might increase de novo epigenetic variation as a source of phenotypic traits and plasticity in response to the new environment and then could undergo natural selection while spreading through its invasive range, resulting in epigenetic differentiation from the EU group and convergence with the native group. This hypothesis needs more evidences in the further study.

Unlike many invasive clonal species spreading mostly by vegetative reproduction (Gao, Geng, Li, Chen, & Yang, 2010; Lambertini et al., 2010; Richards et al., 2012), the invasion of *P. australis* in North America contributes to a reproduction strategy combining sexual and vegetative propagation (Albert, Brisson, Belzile, Turgeon, & Lavoie, 2015; McCormick, Kettenring, Baron, & Whigham, 2010). The high genetic diversity in *P. australis* makes it more difficult to detect the epigenetic changes during the invasion. The epigenetic variation in natural populations can originate from genetic factor (B1), spontaneous epimutations (B2), and environmentally induced epigenetic changes (B3) (Richards et al., 2017). In addition, epigenetic change can create novel genetic variation through regulating transposable elements activity (Richards et al., 2017). In our study, the similarity of genetic and epigenetic structure reflects the drivers B1 and B2. While the structure of B1 and B2 can only be shaped through natural selection, B3 could be direct changed by the environment. The epigenetic differentiation was observed in contrast habitats without variation differentiation (Foust et al., 2016; Gugger et al., 2016; Lira‐Medeiros et al., 2010; Schulz et al., 2014), but it remained unexplored how stable and inheritable the B3 variation is in these studies. Common garden filtered the plastic B3 variation in our study, and epigenetic divergence between populations in the introduced region and in its original region was weakened. Moreover, the heterogeneity of microhabitats within each region can increase epigenetic noise masking the direction of epigenetic changes during the invasion. Finally, it is hard to find the few important adaptive loci using a limited number of anonymous loci provided by AFLP and MS‐AFLP (Schrey et al., 2013). For the above reasons, we failed to detect the significant epigenetic divergence independent of genetic variation.

However, the adaptation of introduced groups cannot be explained entirely by adaptive evolution based on genetic or epigenetic variation in this study. Only a few diverged ecophysiological functional traits of the Mediterranean *P. australis* M1 lineage were detected between the DELTA and MED groups (Guo et al., 2016), and we determined very little differentiation in genetic and epigenetic variation between them. Given that the climate in the introduced range was more advantageous than that in the original range for the survival of common reed because of abundant precipitation and warm temperature, the introduced environment did not provide enough selection pressures to shape the stable genetic and epigenetic structure of introduced populations. Therefore, we cannot ignore the potential effect of preadaptation and ecological fitting based on the inherently high phenotypic plasticity during the invasion process of common reed (Guo, Lambertini, Nguyen, Li, & Brix, 2014; Guo et al., 2016), which may be correlated with plastic epigenetic mechanisms (Gao et al., 2010).

## 5 CONCLUSION

In conclusion, epigenetic variation in introduced groups of *P. australis* is often correlated with genetic variation, suggesting the closely correlated effect of genetic and epigenetic variation in species with high genetic diversity. When the introduced population cannot acquire adequate genetic diversity through hybridization with other introduced populations, the importance of epigenetic variation may rapidly emerge. Faster stable epigenetic convergence between introduced and native groups was not observed maybe due to the strong genetic effect. Further study in situ and in a common garden for several generations is necessary to associate the inheritable genetic and epigenetic loci with invasive traits, and it is better to use a reduced representation bisulfite sequencing approach based on high‐throughput sequencing technology (Robertson & Richards, 2015) for direct comparison with functional genetic context.

## CONFLICT OF INTEREST

None declared.

## AUTHOR CONTRIBUTIONS

LL, WG, and XG conceived the idea and designed the study. WG and XG transplanted reeds from the field and the cooperation garden to our common garden. SL, ND, and CP contributed to garden management and sample collection. LL and CP performed the molecular laboratory work. LL, ND, and XG analyzed and interpreted the data. LL drafted the manuscript, and all authors participated in manuscript modifications and gave final approval for publication.
